# The challenge of long-term stroke outcome prediction and how statistical correlates do not imply predictive value

**DOI:** 10.1093/braincomms/fcaf003

**Published:** 2025-01-23

**Authors:** Christoph Sperber, Laura Gallucci, Marcel Arnold, Roza M Umarova

**Affiliations:** Department of Neurology, Inselspital, University Hospital Bern, University of Bern, 3010 Bern, Switzerland; Department of Neurology, Inselspital, University Hospital Bern, University of Bern, 3010 Bern, Switzerland; Department of Neurology, Inselspital, University Hospital Bern, University of Bern, 3010 Bern, Switzerland; Department of Neurology, Inselspital, University Hospital Bern, University of Bern, 3010 Bern, Switzerland

**Keywords:** imaging biomarker, lesion mapping, recovery, machine learning, disconnection

## Abstract

Personalized prediction of stroke outcome using lesion imaging markers is still too imprecise to make a breakthrough in clinical practice. We performed a combined prediction and brain mapping study on topographic and connectomic lesion imaging data to evaluate (i) the relationship between lesion-deficit associations and their predictive value and (ii) the influence of time since stroke. In patients with first-ever ischaemic stroke, we first applied high-dimensional machine learning models on lesion topographies or structural disconnection data to model stroke severity (National Institutes of Health Stroke Scale 24 h/3 months) and functional outcome (modified Rankin Scale 3 months) in cross-validation. Second, we mapped the topographic and connectomic lesion impact on both clinical measures. We retrospectively included 685 patients [age 67.4 ± 15.1, National Institutes of Health Stroke Scale 24 h median(IQR) = 3(1; 6), modified Rankin Scale 3 months = 1(0; 2), National Institutes of Health Stroke Scale 3 months = 0(0; 2)]. *Predictions* for acute stroke severity (National Institutes of Health Stroke Scale 24 h) were better with topographic lesion imaging (*R*² = 0.41) than with disconnection data (*R*² = 0.29, *P* = 0.0015), whereas predictions at 3 months (National Institutes of Health Stroke Scale/modified Rankin Scale) were generally close to chance level. In the analysis of lesion-deficit *associations*, the correlates of more severe acute stroke (National Institutes of Health Stroke Scale 24 h > 4) and poor functional outcome (modified Rankin Scale 3 months ≥ 2) were left-lateralized. The lesion location impact of both variables corresponded in right-hemisphere stroke with peaks in primary motor regions, but it markedly differed in left-hemisphere stroke. Topographic and disconnection lesion features predict *acute* stroke severity better than the *3-months* outcome. This suggests a likely higher impact of lesion-independent factors in the longer term and highlights challenges in the prediction of global functional outcome. Prediction and brain mapping diverge, and the existence of statistically significant associations—as here for 3-months outcomes—does not imply predictive value. Routine neurological scores better capture left- than right-hemispheric lesions, further complicating the challenge of outcome prediction.

## Teaser

Sperber *et al*. investigated the relationship between stroke lesion features and stroke outcome in a combined brain mapping and prediction study. Predictions were better for acute than for 3-months outcome measures and for topographic than for diconnectomic features. The existence of significant lesion-deficit associations did not imply predictive value.

## Graphical Abstract

**Graphical Abstract:**
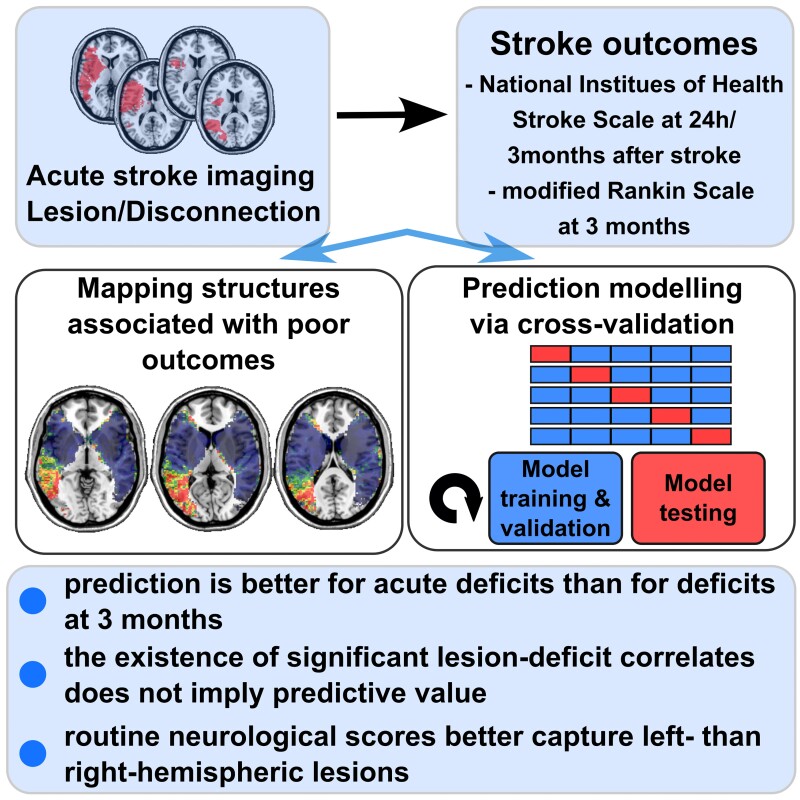

## Introduction

Advances in stroke imaging and prediction algorithms have raised the prospect of clinical application of cognitive neuroscience method repertoires^1-3^: an algorithm that can predict *chronic* stroke outcome and cognitive deficits from *acute* brain imaging markers could be used to tailor individualized therapeutic approaches and guide patient care. To date, however, such algorithms exist only in the context of scientific works and do not yet provide sufficiently good predictive performance for clinical application. In the scientific approach to this challenge, we see two related major pitfalls that impede progress: First, the concept of prediction is often confused with the concept of (statistical mapping of) neural correlates.^4^ Second, the term ‘prediction’ is often used when anatomo-clinical correlates—like in brain mapping—are found *within a sample*, as opposed to the *out-of-sample* prognosis of observations that were not used within the training data set.^5^ Any set of data points can be described with a sufficiently complex model. Thereby, models can be over-fitted to data,^6^ meaning that the model is overly specifically fitted to the training data and poorly suited to predict new data. These problems may be particularly prominent for measures of overall stroke outcome.

The general impact of stroke on function is often evaluated in the early phase as *stroke severity*, mainly assessed with the National Institutes of Health Stroke Scale (NIHSS),^7^ or in the late subacute/chronic phase as *functional outcome*, mainly assessed with the modified Rankin Scale (mRS).^8^ These two measures are frequently used as outcome measures in clinical trials.^9-13^ Our current knowledge to what extent well-defined neurobiological correlates relate to and explain these measures is based on studies that identified topographic or region-wise lesion correlates,^14-17^ including a study that directly compared stroke severity and functional outcome.^14^ On the other hand, some studies predicted the impact of stroke from lesion imaging data.^18-21^ These studies were often limited to within-sample associations, contrary to out-of-sample validation to assess the actual predictive value of a model and anatomical data. Hence, insights into potential lesion imaging markers are limited by the blurred distinction between prediction and mapping. Moreover, previous studies often used low-dimensional predictive models with a small set of only one or a few *a priori* chosen anatomical features. However, high-dimensional models on more complex imaging data were already successfully used to predict specific post-stroke deficits.^2,22,23^

Besides, the question remains whether lesion location adequately explains stroke outcome or if a perspective on network effects is better suited. Particularly measures that indirectly estimate how a lesion disrupts the brain connectome^23,24^ suggest themselves as efficient and easily accessible biomarkers. Previous studies found such measures to explain deficits in specific cognitive and behavioural domains of post-stroke function.^3,23,25^

In the current study, we aimed to provide a comprehensive evaluation of the acute and longitudinal lesion impact on stroke severity and functional outcome with a combined prediction and brain mapping study.

## Materials and methods

### Rationale of the study

The current study addressed methodological and conceptual questions about the relationship between acute stroke lesion imaging and clinically widely used global measures of stroke outcome, namely the NIHSS and mRS. Central to the study is the differentiation between inferential analysis when mapping post-stroke deficits and the prediction of post-stroke deficits. Common lesion-deficit mapping techniques fit a single model for each brain imaging feature to identify brain structures associated with a deficit, meaning that they address purely inferential questions. For the prediction of post-stroke deficits, ideally, models are evaluated out-of-sample, i.e. a model is fit to one set of data and then tested in another sample, e.g. through cross-validation or by using a distinct second sample. With such out-of-sample testing, the design shows how the algorithm would perform on unknown data, i.e. it mirrors the task of an actual prediction algorithm. In summary, both approaches model the relationship between imaging data and clinical variables, but they follow conceptually very different strategies. We would like to note that we use the term ‘*prediction*’ here also for out-of-sample modelling of acute stroke severity based on lesion anatomy. We do so to distinguish models that are tested out-of-sample from within-sample models as used in brain mapping. We performed all analyses both with lesion images or disconnection markers derived from lesion images, as disconnection markers might better represent the neural correlates of post-stroke deficits.^3,23,25^ Our main questions were: (i) How does lesion imaging predict stroke outcome as a function of time since stroke, i.e. when outcomes are acquired in the acute phase or 3 months post-stroke? (ii) How do the statistically mapped neural correlates of stroke outcome vary with the type of outcome measure and time since stroke? (iii) What is the predictive value of lesion imaging features versus derived disconnection measures?

First, we used high-dimensional machine learning models to predict stroke severity/outcome in an out-of-sample prediction analysis. We compared (i) predictions using topographic versus connectomic lesion data and (ii) prediction models for acute stroke severity versus stroke severity and functional outcome at 3 months in another. Second, we captured the associations of lesion location with stroke severity and functional outcome by topographic and disconnection-wise statistical mapping.

### Patients and clinical assessment

We retrospectively analysed clinical routine data of patients with a first-ever ischaemic anterior circulation stroke admitted to the Bern Stroke Centre between January 2015 and October 2020. Only patients with an available MRI acquired about 24 h after admission were included. Patients with a recorded pre-stroke disability (pre-stroke mRS of >1) were excluded. Patients with a recurrent stroke within 3 months were excluded because these cases did not represent the longer-term consequences of the first stroke. A detailed flowchart of exclusions is shown in Supplementary Fig. 1. Written informed institutional general consent for research was available from all participants or their guardians, and the study received approval from the local ethical board (Kantonale Ethikkommission Bern KEK 2020-02273).

Clinical data were recorded by the attending physician and included stroke severity as assessed by the NIHSS^7^. The current study did not focus on the success of reperfusion therapy but on the functional impact of the final stroke lesion. Hence, we utilized the NIHSS at 24 h after symptom onset as a measure of acute stroke severity as it better reflects the final lesion load in patients that undergo acute stroke therapy than NIHSS at admission. Functional outcome was assessed by the mRS^8^ at 3 months after stroke and was either recorded within clinical follow-up examination or by phone. We dichotomized the non-continuous variable to make it suitable for a classification algorithm into favourable (mRS 0–1) versus poor outcome (mRS ≥2). This is a commonly used cut-off for the mRS which,^26^ compared to the other common cut-off of mRS ≥3, created relatively equally sized groups, which is required to train an unbiased classifier. In patients with clinical follow-up, the NIHSS at 3 months was additionally available. The use of the NIHSS 3 months after stroke does not follow the original intention of the scale to measure acute stroke severity but allows a high degree of comparability with the NIHSS measured acutely.

### Brain imaging and lesion segmentation

We created binary lesion masks based on diffusion-weighted MRI acquired at a slice thickness of 4–5 mm with a 1.5T or 3T Siemens scanner about 24 h after stroke onset. We segmented lesions as described previously^27^ by a semiautomatic algorithm using a region-of-interest toolbox in SPM12 (http://www.fil.ion.ucl.ac.uk/spm/software/spm12). We manually selected intensity thresholds for each patient to find the best match between the lesion mask and the hyperintense diffusion-restricted brain tissue. If required, consultation with other available sequences (e.g. FLAIR) assured accurate lesion delineation. Lesion masks were then normalized to the Montreal Neurological Institute space at 2 × 2 × 2 mm³ resolution with normalization parameters from the co-registered T1 scans.

### Estimation of lesion-induced white matter disconnection

We indirectly estimated the disconnection of white matter fibres with the Lesion Quantification Toolkit.^28^ The concept behind the indirect estimation of white matter disconnection is to overlay a patient’s lesion mask with a reference white matter streamline atlas obtained in healthy subjects—here the HCP-842 population-averaged streamline tractography atlas^29^—and identify white matter streamlines assumed to be disconnected by the lesion.^30^

We resliced lesion masks to 1 × 1 × 1 mm³ resolution and processed them with the Lesion Quantification Toolkit. We estimated the parcellation-based region-to-region disconnection by assessing fibre streamlines of the healthy reference connectome that intersect a patient’s lesion mask. Next, the end points of each streamline were identified and assigned to grey matter regions defined by the BN246 human Brainnetome Atlas,^31^ which is a multimodal connectivity-based parcellation of 246 grey matter regions. For each patient, the number of disconnected streamlines between each possible pair of grey matter regions was recorded. The final output was a symmetric 246-by-246 disconnection matrix providing the count of disconnected streamlines for each pair of brain regions.

### Statistical analysis

#### Assessment of the predictive value of lesion topographies and disconnection

**Figure 1:**
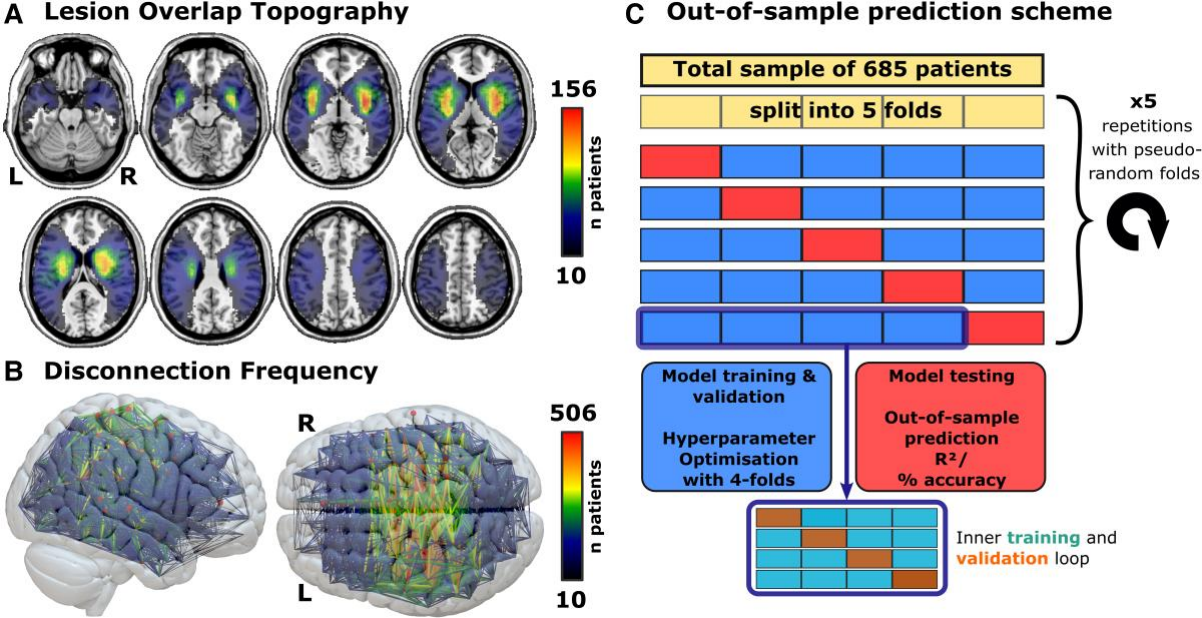
**Lesion topography and cross-validation scheme**. **(A)** Lesion overlap topography of all 685 lesions after normalization. **(B)** Edge-and-node plot indicating the number of patients with at least one disconnected streamline between each pair of nodes. **(C)** Nested cross-validation scheme to assess model generalizability in out-of-sample predictions (adapted and modified from^32^). Supplementary Fig. 4 shows colourblind-friendly versions of all relevant subplots of all figures.

We processed both lesion topographies (i.e. the binary lesion masks) and disconnection matrices to optimize their applicability in prediction algorithms. We first removed features with little to no variance by masking voxels lesioned in less than 10 patients (Fig. 1A) and region-to-region connections with disconnection in less than 10 patients (Fig. 1B), as well as redundant features in the symmetric disconnection matrix. The remaining 93 547 voxels and 2959 region-to-region connections were used as the first set of predictors. Additionally, we derived a lower-dimensional componential feature space by principal component analysis, which is an efficient way to use high-dimensional lesion imaging data,^33^ and retained components that explained at least 1% of the total data variance. All models additionally contained the variable age which impacts stroke outcome,^34^ but we did not include lesion volume, which does not improve models that already account for lesion location.^32^ Continuous predictors were normalized. NIHSS values were skewed; hence, we log-transformed this variable and re-transformed the predictions before the final evaluation.

The out-of-sample prediction performance of several machine learning algorithms was evaluated in a nested 5-fold cross-validation scheme (Fig. 1C; adapted from^35^). Models were trained and validated by cross-validation in the four inner folds, which included hyperparameter optimization. Only after model optimization, the out-of-sample prediction in the remaining test fold in the outer loop was obtained. Repeating this procedure through all 5 folds as the outer fold generated an out-of-sample prediction for each patient. We then repeated the entire nested procedure five times to control for fluctuations introduced by the fold assignment. Each of the five repetitions used a pseudo-randomized assignment to folds that we kept constant across all conditions. The final prediction for each patient was the average predicted score respectively the majority decision across all five repetitions. Hence, each estimate of prediction performance originated from 25 repetitions of the modelling procedure. We evaluated the final model performance by the coefficient of determination *R*² and Pearson’s correlation between actual and predicted scores for continuous variables, and the classification accuracy, i.e. the proportion of correct classifications, as well as positive and negative predictive values (PPV/NPV) for categorical variables. We statistically compared performance between models by comparing regression residuals with a paired *t*-test respectively classification decisions with a McNemar test. All analyses were scripted in Matlab R2022b with the regression learner toolbox. For the main analyses, we included stroke patients with all degrees of initial stroke severity, including those with minor strokes, to enable the algorithms to learn where brain lesions have little effect on outcome or can be compensated. However, we also performed control analyses that more closely approximate the application of a potential prognostic algorithm in the clinical setting. For the best-performing algorithms in the main analyses, we repeated (i) all predictions while excluding subjects with very low or no initial deficits (=minor stroke, NIHSS at 24 h ≤ 1), i.e. patients for which a prognosis would not be needed in a clinical setting, and (ii) with baseline stroke severity (i.e. NIHSS at 24 h) as an additional predictor in models of deficits at 3 months.

#### Prediction algorithms

For the regression of NIHSS 24 h, we used *support vector regression* (SVR) and *Gaussian process regression* (GPR). For the classification of favourable versus poor functional outcome according to the mRS at 3 months, we used *support vector machines* (SVM) and bagged decision trees as *random forests* with 100 trees. Hyperparameters were optimized by Bayesian optimization. Details on model parameters and hyperparameters are provided in the Supplementary Materials.

In an additional analysis on the subsample of patients with an NIHSS at 3 months available, we compared the prediction performance for NIHSS at 24 h versus 3 months post-stroke using the regression algorithm that performed best for NIHSS at 24 h in the total sample.

#### Direct comparison of prediction value for acute stroke severity and functional outcome at 3 months

To directly compare the predictive value of the imaging data between acute stroke severity versus late subacute functional outcome, we binarized acute stroke severity (NIHSS 24 h) so that both variables could be predicted in a comparable classification design. Further, we stratified the positive base rate of both variables to level the prediction chance level. We found that a binarization into mild stroke at NIHSS ≤4 and non-mild stroke at NIHSS >4 was best suited to stratify our data. We further randomly removed 28 patients so that both NIHSS and mRS were more pathological (i.e. non-mild NIHSS/unfavourable functional outcome) in the exactly same proportion of patients (270/657 = 41.1%). Importantly, although both binary variables corresponded often (70.8%), they were still significantly different (*χ*²(1) = 103.2; *P* < 0.0001). The additional prediction analyses were restricted to the models that performed best in the previous analysis. We evaluated associations between the target variable (NIHSS/mRS), imaging data type (lesion topography/disconnection), and prediction accuracy (true/false) using log-linear models. Specifically, we performed likelihood ratio tests to compare models with different interaction structures. First, we compared a model that included an interaction between the target variable and accuracy (target variable * accuracy + imaging data type) with a simpler model that assumed no interaction (target variable + accuracy + imaging data type). Second, we tested whether an interaction between the target variable and imaging data type improved the fit by comparing a model that included both interactions (target variable * accuracy + imaging data type * accuracy) to the saturated model, which incorporated all possible interactions (target variable * accuracy * imaging data type).

#### Mapping the location impact score of higher stroke severity and poor functional outcome

We mapped the topographic and disconnectomic neural correlates of mild versus non-mild stroke severity (NIHSS 24 h ≤ 4 versus >4) and favourable versus poor functional outcome (mRS 3 months <2 versus ≥2). To maximize the comparability between both variables, we analysed stratified samples with equal rates of higher stroke severity and poor functional outcome, as described in the previous section. For each voxel or disconnection, we mapped the association between the feature’s pathology status and the binary clinical variable either with exact Fisher tests for lesion data or with logistic regression for disconnection data. The corresponding odds ratios were mapped across brain voxels to determine a voxel-wise lesion impact score. We controlled for multiple comparisons by a false-discovery rate (FDR) correction with *q* = 0.01. For each Brodmann area (BA) for which we tested at least 50 voxels, we additionally assessed the median odds ratio. BAs were defined by the atlas in MRIcron (https://www.nitrc.org/projects/mricron).

For the subsample of patients with NIHSS at 3 months available, we directly compared the neural correlates of NIHSS at 24 h versus NIHSS at 3 months. With both variables being continuous, we used two-sample *t*-tests controlled for multiple comparisons by a FDR correction with *q* = 0.01. We mapped the mean difference between groups as an effect size to determine a lesion impact score.

## Results

The final sample included 685 patients for which demographic and clinical data are shown in Table 1. The distributions of stroke severity and functional outcome are shown in Supplementary Fig. 2. In total, 298 out of 685 patients (43.5%) had a poor functional outcome as indicated by mRS of ≥2. For a subsample of 306 patients, the NIHSS at 3 months was available [median(IQR) = 0(0; 2); 46.7% patients with an NIHSS 3 months > 0]. The distributions of stroke severity at 24 h and 3 months in this subsample are shown in Supplementary Fig. 3.

**Table 1:** Demographic and clinical data

	All participants, *N* = 685	Subsample, *N* = 306
Age, years mean (SD; range)	67.4 (15.1; 18–95)	66.0 (14.5; 19–94)
Sex, Male %	56.5	58.5
History of transient ischaemic attack, %	4.3	2.6
Hypertension, %	67.3	64.1
Diabetes, %	15.1	14.7
Smoking, %	28.5	27.5
Hyperlipidaemia, %	69.3	68.6
Atrial fibrillation, %	26.0	21.9
Coronary heart disease, %	16.1	14.7
NIHSS 24 h, median(IQR)	3 (1; 6)	3 (1; 5)
NIHSS 3months, median(IQR)	–	0 (0; 2)
mRS 3 months, median(IQR)	1 (0; 2)	1 (0; 2)
Lesion size in cm³, median(IQR)	19.1 (5.2; 51.6)	19.9 (5.2; 45.5)
Stroke lateralization Left/Right, %	54.5; 45.5	62.1; 37.9

Risk factors included missing values (not more than 4.8% of the total sample) which were omitted in the computation of characteristics. NIHSS at 3 months was computed for a subsample of 306 patients. Lesion size was computed based on the MNI-normalized lesion map. SD, standard deviation; NIHSS, National Institutes of Health Stroke Scale; mRS, modified Rankin Scale.

The NIHSS 24 h and at 3 months were highly correlated (*τb* = 0.51, *P* < 0.0001), the NIHSS 24 h and the mRS at 3 months were moderately correlated (*τb* = 0.41, *P* < 0.0001) and the NIHSS and the mRS at 3 months were highly correlated (*τb* = 0.58, *P* < 0.0001).

### Predictive value of lesion topographies and disconnection

Detailed prediction results, including the performance of the runner-up models, are shown in Supplementary Table 1. For acute stroke severity (NIHSS 24 h), lesion topographies predicted 40.9% of the variance (SVR on componential data, *R*² = 0.409; Pearson’s *r* = 0.67), whereas structural disconnection only predicted 29.2% of the variance (SVR on componential data, *R*² = 0.292; Pearson’s *r* = 0.58). The lesion topographies were significantly better at predicting stroke severity than disconnection data (*t*(684) = 3.19; *P* = 0.0015).

For prediction of favourable (mRS 3 months < 2) versus poor (mRS 3 months ≥2) functional outcome, lesion topographies achieved a classification accuracy of 64.8% (SVM on voxel-wise data; chance level 56.6%; PPV = 0.74; NPV = 0.62). Disconnection data demonstrated a classification accuracy of 59.9% (SVM on full inter-regional disconnection data; PPV = 0.62; NPV = 0.60), which was significantly inferior to the classification with lesion topographies (*P* = 0.0011).

In the subsample of 306 patients with an NIHSS at 3 months available, we repeated the prediction for both NIHSS at 24 h and 3 months with the algorithm that provided the numerically best performance in the prediction of NIHSS 24 h in the total sample. Lesion topographies predicted 28.2% of variance for the NIHSS 24 h (*R*² = 0.282; Pearson’s *r* = 0.57) but almost no variance for the NIHSS at 3 months (*R*² = 0.006; Pearson’s *r* = 0.31). Disconnection data predicted 22.9% of the variance for the NIHSS at 24 h (*R*² = 0.229; Pearson’s *r* = 0.54) and no variance for the NIHSS at 3 months (*R*² = 0; Pearson’s *r* = 0.24).

### Control analyses

We performed control analyses to evaluate the prediction performance under exclusion of patients with low acute stroke severity (NIHSS 24h ≤ 1, i.e. minor stroke) and including acute stroke severity (NIHSS 24 h) as an additional predictor for prediction of 3 months outcome.

Out of the total sample of 685 patients, 441 had an NIHSS 24 h > 1. For these 441 patients, the prediction of poor stroke outcome (mRS ≥2) without additional inclusion of NIHSS 24 h achieved a classification accuracy of 55.3% with lesion data (SVM on componential data; chance level 54.7%; PPV = 0.57; NPV = 0.51) and 54.7% with disconnection data (SVM on the counts of disconnected streamlines; PPV = 0.55; NPV = 0.50). With acute stroke severity (NIHSS 24 h) as an additional predictor, the accuracy was significantly improved both for lesion data (McNemar’s test *P* = 0.004) with an accuracy of 64.2% (SVM on componential data; PPV = 0.72; NPV = 0.58) and for disconnection data (*P* = 0.016) with an accuracy of 62.8% (SVM on componential data; PPV = 0.68; NPV = 0.58).

Out of the subsample of 306 patients for which an NIHSS at 3 months was assessed, 190 had an NIHSS 24 h > 1. For these 190 patients, acute stroke severity (NIHSS 24 h) could be predicted with *R*² = 0.193 (Pearson’s *r* = 0.40) from lesion data and with *R*² = 0.124 (Pearson’s *r* = 0.19) from disconnection data. The prediction of subacute stroke severity (NIHSS 3 months) explained no variance without acute stroke severity as an additional predictor (*R*² = 0 both for lesion and disconnection data). With acute stroke severity as an additional predictor, the models achieved *R*² = 0.033 for lesion data and *R*² = 0.034 for disconnection data.

### Direct comparison of prediction of acute stroke severity and functional outcome

We directly compared the prediction of acute stroke severity (NIHSS 24 h) and functional outcome at 3 months (mRS 3 months) with a comparable classification algorithm, for which the sample was stratified to contain the same base rates of higher stroke severity and poor outcome. In this stratified sample of 657 patients, the prediction of mild (NIHSS 24 h ≤ 4) versus non-mild acute stroke severity (NIHSS 24 h > 4) was more accurate than the prediction of favourable (mRS < 2) versus poor functional outcome (mRS ≥ 2; (*χ*²(2) = 15.21; *P* < 0.001). The log-linear model with the interaction between imaging data type (lesion topography/disconnection) and variable (NIHSS 24 h/mRS) was not superior to the model without the interaction (*χ*²(1) = 0.12; *P* = 0.94), meaning that predicting acute stroke severity was equivalently better than predicting functional outcome for both lesion topographies and disconnection data. Prediction accuracies are shown in Table 2.

**Table 2:** Results of the direct comparison between prediction of acute stroke severity and late subacute functional outcome

NIHSS 24 h	Acc.	PPV	NPV
SVM lesion (pca)	74.0%	0.74	0.74
SVM disconnection (full)	72.0%	0.72	0.72
mRS 3 months			
SVM lesion (full)	67.7%	0.79	0.66
SVM disconnection (full)	64.2%	0.68	0.64

Out-of-sample classification accuracies in the stratified sample for prediction models based on either lesion or disconnection data. Only the performance of the best model is shown here. ‘full’ denotes conditions where the entire feature set was superior, and ‘pca’ conditions in which the feature space reduced by principal component analysis was superior. Chance level was 58.9%. Acc., classification accuracy; NPV, negative predictive value; PPV, positive predictive value; SVM, support vector machine.

### Lesion location impact on stroke severity and functional outcome

**Figure 2:**
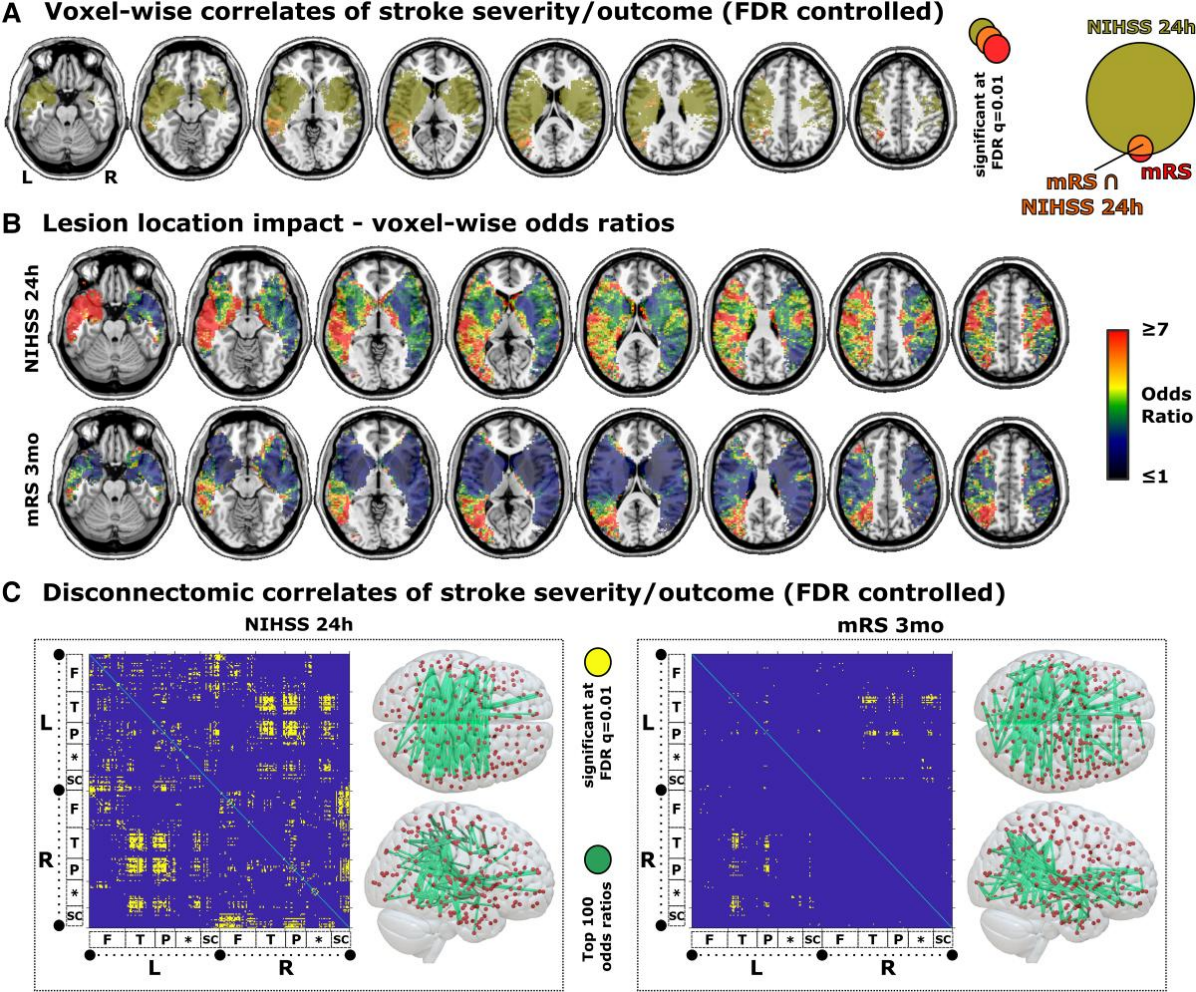
**Neural correlates of higher acute stroke severity and poor functional outcome**. Results of the anatomo-clinical mapping of mild versus non-mild acute stroke severity and favourable versus poor functional outcome at 3 months with the full sample of 685 patients. **(A)** Corrected results of the statistical mapping with exact Fisher’s tests for topographical data. The Venn diagram visualizes the intersection of the neural correlates after false-discovery rate (FDR) correction. **(B)** Corresponding voxel-wise odds ratios. **(C)** Corrected results for logistic regression on connectomic data visualized as symmetric 2D disconnection plots. The 3D plot visualizes the 100 connections with the highest associations. 2D disconnection plots show disconnectivity profiles for left (L) and right (R) hemisphere areas in the frontal lobe (F), temporal lobe (T), parietal lobe (P), the insular, limbic or occipital lobe (*) and subcortical nuclei (SC). See the Supplementary Materials for detailed information. NIHSS, National Institutes of Health Stroke Scale; mRS, modified Rankin Scale.

The neural correlates of non-mild versus mild acute stroke severity—i.e. the binarized NIHSS at 24 h at >4 versus ≤4) were more widespread than the neural correlates of poor versus favourable functional outcome both for the topographic and disconnectomic data (Fig. 2A). For topographic data, the brain regions with the highest lesion impact on higher stroke severity (NIHSS 24 h > 4, Fig. 2B) were strongly left-lateralized and included the left temporal lobe, the left temporo-occipital region, left posterior frontal regions, bilateral precentral gyri and bilateral corticospinal tracts. Poor functional outcome (mRS ≥2) was associated with a much smaller area (∼40 cm³) that was localized mainly in the left posterior middle cerebral artery territory, including the left middle temporal gyrus, the temporo-parieto-occipital region, the inferior parietal lobe and periventricular white matter. The topographic odds ratios across BAs are shown in Supplementary Table 2. Lesions to the right primary motor cortex (BA4) had the highest impact on both variables. In the left hemisphere, the odds ratios were generally higher and the most strongly implicated regions differed markedly between stroke severity and functional outcome.

We *post hoc* assessed the hemispheric discrepancy by a correlation analysis of the voxel-wise odds ratios between higher acute stroke severity and worse functional outcome. We found a medium correlation of odds ratios between NIHSS 24 h and mRS 3 months in the right hemisphere [*r*(46 093) = 0.308; *P* < 0.0001; 95% CI = (0.300; 0.317)], but only a very low correlation in the left hemisphere [*r*(47 450) = 0.068; *P* < 0.0001; 95% CI = (0.059; 0.077)].

On the level of disconnections (Fig. 2C), an extensive, brain-wide connectome of ∼2600 connections was implicated for higher (NIHSS 24h > 4) versus mild (NIHSS 24 h < 4) acute stroke severity. For poor versus favourable stroke outcome at 3 months, 314 mostly interhemispheric connections were implicated which were entirely included as a subset in the results for the NIHSS. The regions with the most implicated disconnections for the mRS in descending order were the left caudal inferior parietal lobule, the left rostroventral inferior parietal lobule, the left nucleus accumbens, the left rostroventral inferior parietal lobule and the right medial superior occipital gyrus.

**Figure 3:**
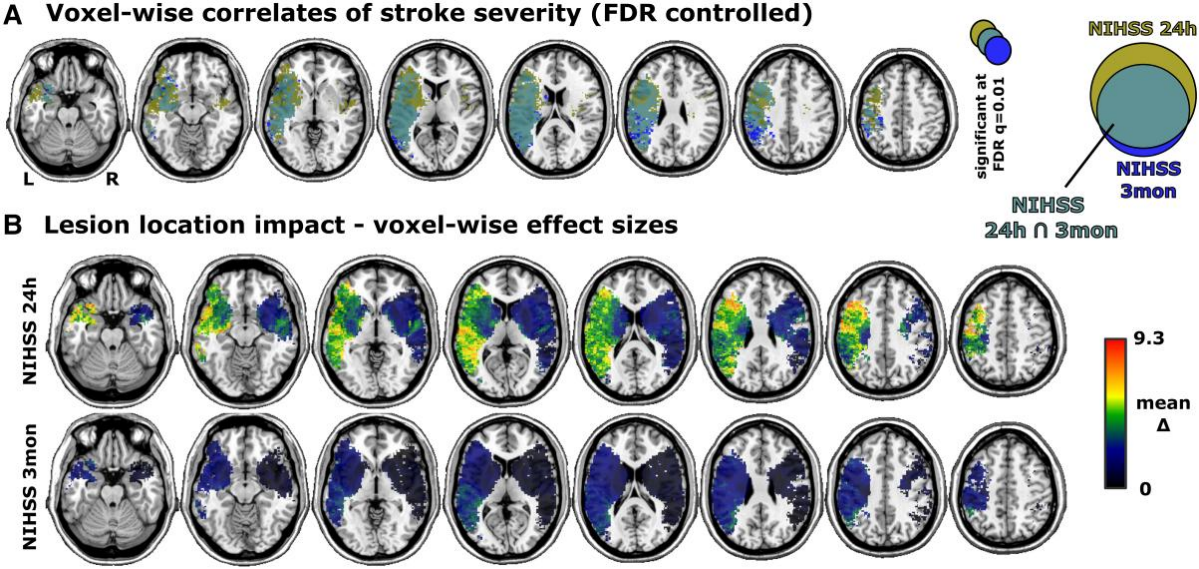
**Neural correlates of stroke severity measured with NIHSS at 24 h and 3 months**. Results of the anatomo-clinical mapping of stroke severity in the subsample of 306 patients with two-sample *t*-tests on continuous data. **(A)** Corrected results of the statistical mapping analyses for topographical data 24 h and 3 months post-stroke. Indicated voxels are significantly associated with higher stroke severity. The Venn diagram visualizes the intersection of the neural correlates after correction. **(B)** Maps of voxel-wise effect sizes as assessed by the difference of means between patients with a lesion in the voxel versus without a lesion in the voxel. Dark values also represent some fluctuation around 0 with negative effect sizes.

Finally, we compared the voxel-wise statistical mapping of stroke severity measured continuously using NIHSS both 24 h and 3 months post-stroke. In the subsample of 306 patients with the NIHSS at 3 months available, the neural correlates of higher stroke severity at 24 h were strongly left-lateralized (Fig. 3A). The correlates of higher stroke severity at 3 months were fewer but included areas of the left inferior parietal lobe that were not implicated for stroke severity at 24 h. The lesion impact topographies (Fig. 3B) re-iterated the higher impact of left-hemispheric lesions. In line with the findings on functional outcome, lesions in the left temporo-parieto-occipital region were again associated with the highest NIHSS scores at 3 months. Of note, any differences in the acute neural correlates of higher stroke severity between the analyses shown in Fig. 2 and Fig. 3 might not only originate from different variable scaling (binary versus continuous) but also varying statistical power due to different sample sizes.

## Discussion

In a combined prediction and brain mapping study, we investigated the topographic and connectomic neural correlates of the acute and late subacute impact of stroke and their predictive value as biomarkers. We found that topographic lesion data better predicted acute stroke severity at 24 h than functional outcome or stroke severity at 3 months. In line with this finding, lesion location impact scores were stronger for the acute than for the late subacute impact of stroke. The poor predictive performance for outcomes at 3 months shows that the mere existence of statistical neural correlates does not imply predictive value. The prediction of outcomes at 3 months was significantly improved by adding acute stroke severity as an additional predictor besides the imaging data. Anatomo-clinical correlates as found by brain mapping were strongly left-lateralized and, on the level of odds ratios, corresponded between acute stroke severity and functional outcome in the right hemisphere, with a focus on the primary motor cortex. However, in the left hemisphere, they differed markedly and did not focus on the primary motor cortex for either variable. Further, lesion topographies allowed better predictions than white matter disconnection data.

### Mapping versus prediction—statistical neural correlates and predictive value diverge

Statistical maps of anatomo-clinical correlations are assumed to provide clinically relevant prognostic information.^18,36^ However, the mere existence of statistical anatomo-clinical correlates—i.e. the fact that a variable can be mapped onto the brain—does not imply a predictive value of the anatomical data.^4^ In the current study, the prediction of late subacute outcome 3 months after stroke was inferior to the prediction of acute stroke severity. The prediction of late subacute outcome was numerically close to chance level, both for functional disability assessed by mRS and stroke severity assessed by NIHSS. This finding is in stark contrast to the results of the statistical mapping analyses, as statistically significant neural correlates were identified for all variables. Most strikingly, we found large neural correlates for stroke severity 3 months after stroke, but the explained variance in the high-dimensional prediction models was close to 0. Similarly, the widespread connectomic neural correlates of acute stroke severity and functional outcome at 3 months did not imply improved predictions. The discrepancy between mapping and prediction becomes intuitively understandable in the face of the requirements that both have. For brain mapping, any association between anatomy and the clinical variable is sufficient to map a function, and, with large samples, even tiny effects can be mapped. For prediction, sensitive and specific predictors are required that do not merely describe the training sample, but that inform about the status of new individual cases. This also implies that the results of brain mapping alone are not necessarily prognostically useful. Statistical brain mapping results may, to some degree, solely reflect the status of small subsamples,^37^ it can be strongly affected by various aspects of the study design,^38^ and may even suffer from methodological biases.^39^

### Acute versus late subacute impact of stroke lesions

The neural correlates of acute stroke severity were widespread and covered large parts of the middle cerebral artery territory with a focus on left-hemispheric areas. Hence, even though subcomponents of stroke severity as measured by the NIHSS have distinct neural correlates,^16^ our results suggest that the overall NIHSS score is affected by lesions in most areas of the middle cerebral artery territory. The same was true for connectomic data, where disconnection of the majority of existing fibre connections was associated with stroke severity. In contrast, worse functional outcome was only significantly associated with lesions to mostly left temporo-parieto-occipital and periventricular lesions. The higher impact of left-hemispheric and especially temporo-parieto-occipital lesions was mirrored in the mapping of the NIHSS at 3 months. Odds ratios for functional outcome also suggested a large effect of right-hemispheric lesions of the primary motor cortex, but not strong enough to create interpretable clusters after statistical control. On the connectomic level, a set of mostly interhemispheric connections was implicated.

Our findings reiterate that anatomo-clinical correlations are larger and more widespread for acute stroke severity than for functional outcome at 3 months.^14^ However, our exact topographic results deviate from previous studies that found statistical peak values focussing on frontal and subcortical areas,^14,19,20,40^ while our results suggested an additional high impact of left temporo-parietal areas. This variability across studies with large sample sizes may be explained by the timing of imaging and neurological assessment, as many clinical variables can highly vary in the acute stage of stroke (e.g. in the first hours versus 24 h after symptoms onset). Likewise, sample characteristics, related e.g. to the designation of a stroke unit and its catchment area, may play a role. The heterogeneity of lesion-deficit correlates across brain regions suggests that the NIHSS and mRS does not reflect the impact of lesions in some areas. Of note, our study only included patients with stroke to the anterior circulation and, therefore, our findings cannot be generalized to patients with lesions in the posterior circulation.

The generally lower lesion location impact on functional outcome, as well as the worse prediction performance, might be partially explained by the higher reliability of the NIHSS over the mRS.^9,41^ Hence, in studies using the mRS as a measure of functional outcome and in the development of high-precision prognostic models in particular, one should be aware of the limitations due to reliability.^42^ Further, the NIHSS explicitly represents a collection of deficits to several different functions, e.g. motor or language functions, and could therefore be related to more extensive brain regions. In contrast, the mRS is a global outcome rating scale of functional disability, also based on pre-stroke activities rather than only the performance observed on a specific task.^41^ However, we likewise found an advantage in the prediction of acute versus late subacute stroke outcome in the direct comparison of NIHSS at 24 h versus 3 months. Hence, another and maybe more substantial explanation is that lesion-independent factors predominantly impact recovery trajectories and longer-term stroke outcome, but less so acute stroke severity. These could be comorbidities or clinical or demographic resilience factors, such as brain and cognitive reserve. While these variables are also known to affect acute post-stroke deficits,^27,43^ their impact might be amplified in the longer term due to their interaction with brain plasticity^44^ and response to rehabilitation. Therefore, imaging data alone may not be sufficient to predict global outcome measures beyond the acute stroke phase, and prognostic precision medicine may particularly depend on knowing and appropriately incorporating these factors into models. In conclusion, the longer-term prognosis of the impact of a stroke lesion on functional disability and neurological impairment with imaging biomarkers appears to be more challenging than the prognosis of the short-term impact on neurological impairment.

### A fundamental difference between the impact of left- and right-hemisphere lesions?

Our results are in line with previous studies that found a larger impact of left-hemispheric lesions on stroke severity^14^ and functional outcome.^14,19,20^ This left-hemispheric focus is not necessarily surprising for stroke severity given that the NIHSS explicitly includes several language tests, but it is so for functional outcome. The NIHSS has already been shown to better capture left-hemispheric than right-hemispheric damage.^45,46^ Besides, the impact of language on activities of daily living, general psychological stress and rehabilitation^47^ might play a role.

Our findings add another layer to the hemispheric differences: although anatomo-clinical associations were generally weaker for functional outcome than for stroke severity, they were similar in right-hemispheric stroke and both focussed on the right primary motor cortex. On the other hand, areas with peak lesion location impact markedly differed between stroke severity and functional outcome in the left hemisphere, and the impact of lesions to the left motor cortex did not stand out as particularly high. Hence, our findings are largely in line with the known relevance of motor function in stroke severity and functional outcome.^48,49^ However, they also suggest a complex influence of other functions specifically in left-hemispheric stroke, which could be language functions.

### Lesion topographies and connectomic data

Lesion location has been criticized for only poorly representing the network architecture of the human brain.^50^ Connectomic measures that either incorporate information about white matter fibres^24,28,51^ or functional connectivity^17,52,53^ were suggested to provide a deeper understanding of the pathology underlying post-stroke deficits. However, the comparison of topographic and connectomic measures as biomarkers provided varying results^22,25,52,54,55^ showing that an explanatory superiority of connectomic measures is specific to certain deficits, especially higher cognitive functions.^52^ This might explain the lower predictive value of white matter disconnection in the current study. With a major role of low-level functions such as motor and language skills as captured by the NIHSS and mRS, lesion topographies might be generally better suited as a biomarker. The question remains if other functional or different structural connectomic measures provide better biomarkers.

### Limitations

The current study utilized dichotomized variables derived from the NIHSS and mRS in several analyses. The dichotomization reduces the informational value of each variable alone and ordinal or continuous models, as, for example, for stroke severity in our first experiment, might better capture clinical variance.^9,18,26^ However, the dichotomization and stratification of both variables allowed us to directly compare the variables both anatomically and regarding their predictive value, irrespective of differences in scaling and data distributions. We defined mRS scores ≥2 as poor outcome, which is a common, but less frequent cut-off than ≥3.^26^ Therefore, the poor outcome category encompassed a wide range of mRS scores. Further, the applied measures of stroke outcome are global ones, and studies that access fine-grain subcomponents of neurological scores, as previously done for the NIHSS,^16^ could provide further insights. On the other hand, exactly these global measures—NIHSS and mRS—are the most widely used stroke outcome measures. We estimated disconnection indirectly, whereas the direct assessment of white matter disconnection with each patient’s diffusion-tensor data might provide better results.

## Supplementary Material

fcaf003_Supplementary_Data

## Data Availability

Analysis scripts, additional materials and statistical maps are available at https://data.mendeley.com/datasets/2s9pkmccx2/1. Original clinical data are not publicly available, but qualified researchers may request access to anonymized data. Proposals need to be approved by the local ethics committee.
