# Supplementary material for: The challenge of long-term stroke outcome prediction and how statistical correlates do not imply predictive value
Source: Brain Commun. 2025 Jan 23;7(1):fcaf003. doi: 10.1093/braincomms/fcaf003 (PMC11756379; doi:10.1093/braincomms/fcaf003)
Supplement: fcaf003_Supplementary_Data [file fcaf003_supplementary_data.pdf]

Supplementary Materials

Details on prediction models

For regression of NIHSS 24h, we used *support vector regression* with a radial basis function kernel and the hyperparameters box constraint C, kernel scale γ, and ε. The box constraint C (also known as soft margin) was chosen among positive values log-scaled in the range [1e-3,1e3]. Kernel scale γ was chosen among positive values log-scaled in the range [1e-3,1e3]. ε was chosen among positive values log-scaled in the range [1e-3,1e2]*IQR(Y)/1.349.

Next, we used *Gaussian process regression* with a rational quadratic kernel and the hyperparameters kernel scale, basis function and σ. Basis function was selected as either 'constant', 'none', 'linear', or 'pure quadratic'. Kernel scale was chosen among real values in the range [1e-3*MaxPredictorRange, MaxPredictorRange], where MaxPredictorRange = max(max(X) - min(X)). σ was chosen among real values in the range [1e-4, max(1e-3,10*ResponseStd)], where ResponseStd = std(y).

For classification, we used *support vector machines* (SVM) with a radial basis function kernel and the hyperparameters box constraint C, kernel scale γ with the same hyperparameter ranges as for support vector regression. Next, we used bagged decision trees as *random forests* with 100 trees. For each split, sqrt(n) out of n features were randomly considered. We tuned the hyperparameter minimal leaf size by Bayesian optimisation. This limits the minimum number of observations for a node to be still split. This parameter indirectly also limits tree depth, as small nodes (i.e. nodes with only a few observations) are not further split to generate nodes at the next depth level. The parameter was selected between 1 and 30.

Supplementary Table 1: Results of the comparison between topographic and disconnectomic predictors

Out-of-sample prediction performance for models based on either lesion or disconnection data. All models also included the predictor age. 'Full' denotes conditions where the entire feature set was used, and 'pca' denotes conditions in which the feature space was reduced by principal component analysis. The best model in each category is highlighted in bold. R^2 - coefficient of determination; Acc. – Classification accuracy; GPR – Gaussian process regression; SVM – Support vector machine; SVR – Support vector regression.

NIHSS 24 h – Lesion topography	R^2	NIHSS 24h - Disconnection	R^2
SVR (full)	0.387	SVR (full)	0.276
SVR (pca)	**0.409**	SVR (pca)	**0.292**
GPR (full)	0.347	GPR (full)	0.203
GPR (pca)	0.381	GPR (pca)	0.288

mRS – Lesion topography	Acc.	mRS - Disconnection	Acc.
SVM (full)	**64.8 %**	SVM (full)	**59.9 %**
SVM (pca)	64.4 %	SVM (pca)	59.6 %
Random Forest (pca)	62.3 %	Random Forest (pca)	59.7 %

Supplementary Table 2: Detailed results on lesion impact across BA regions

Additional region-wise interpretation of lesion location impact for all BA areas with at least 50 tested voxels. We computed the median odds ratio out of all voxel-wise odds ratio values in the area to assess the region-wide lesion location impact.

	Left hemisphere		Right hemisphere	
BA Area	Median OR NIHSS 24h	Median OR mRS 3 months	Median OR NIHSS 24h	Median OR mRS 3 months
1	5.09	3.10	5.02	2.50
2	4.25	3.23	3.77	2.23
3	5.24	3.49	4.78	2.88
4	6.90	3.84	7.12	4.37
6	4.76	2.65	4.49	2.42
7	4.74	9.49	3.48	2.30
8	5.20	3.99	2.73	2.11
9	6.39	3.99	2.62	2.08
10	2.50	4.44	2.73	3.08
11	5.28	1.68	3.24	3.60
18	6.31	6.31	2.73	0.860
19	5.02	5.84	2.73	1.30
20	10.45	3.99	2.72	2.38
21	7.56	4.54	3.02	2.23
22	6.48	3.68	3.05	2.07
25	6.31	1.92	4.48	2.86
28	11.89	2.20	2.83	2.16
34	7.34	1.86	3.58	2.13
35	6.96	2.66	4.36	2.65
36	13.02	3.32	2.35	2.43
37	5.17	6.31	2.73	1.81
38	10.4	2.42	2.67	2.46
39	4.96	5.08	2.62	1.84
40	4.25	4.08	3.42	2.47
41	4.76	3.18	2.84	2.13
42	5.17	2.98	3.12	2.08
43	7.54	2.48	4.60	3.18
44	4.51	2.17	3.59	1.93
45	4.51	2.08	3.48	1.77
46	5.51	3.53	2.71	2.38
47	3.73	2.07	3.23	2.09
48	4.31	2.14	3.31	1.88

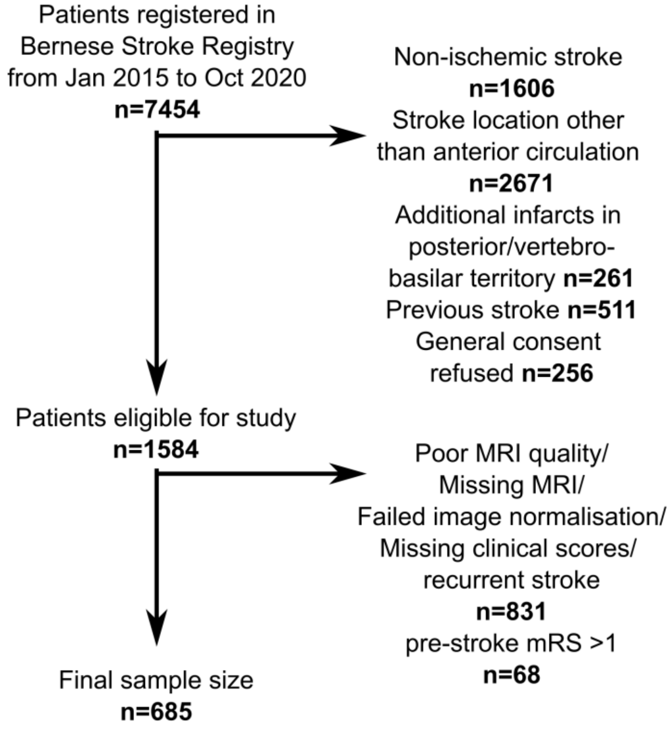

Supplementary Figure 1: Flowchart

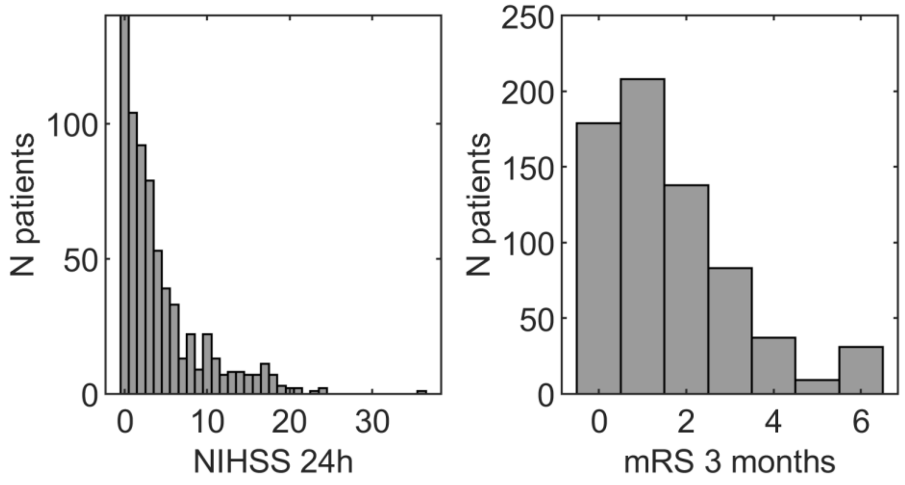

Supplementary Figure 2: Distribution of NIHSS scores at 24h and mRS at 3 months

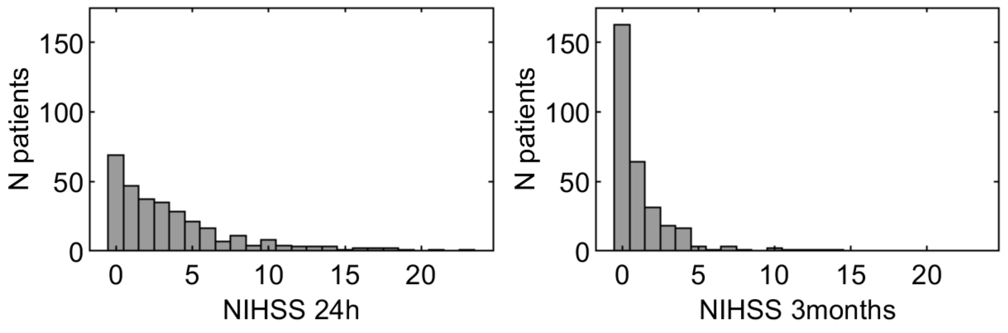

Supplementary Figure 3: Distribution of NIHSS scores at 24h and 3 months

This figure only includes the subsample of 306 patients with an NIHSS at 3 months available.

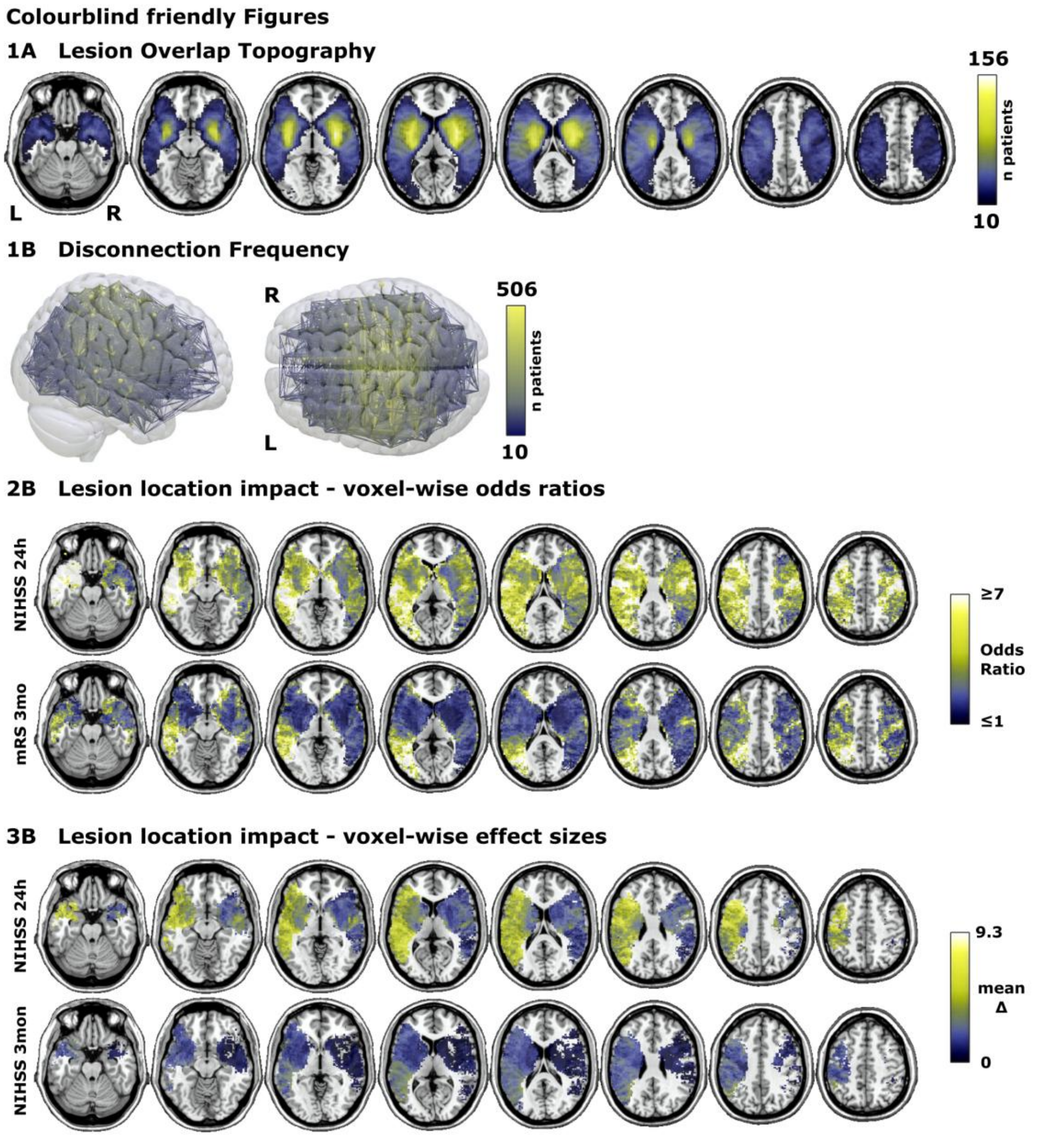

Supplementary Figure 4: Colourblind-friendly version of Figure 1A, 1B, 2B, 3B
